# Multiple factors influence the contribution of individual immunoglobulin light chain genes to the naïve antibody repertoire

**DOI:** 10.1186/s12865-014-0051-2

**Published:** 2014-10-30

**Authors:** Sean P Fitzsimmons, Antonina G Aydanian, Kathleen J Clark, Marjorie A Shapiro

**Affiliations:** Laboratory of Molecular and Developmental Immunology, Division of Monoclonal Antibodies, OBP, CDER, FDA, 10903 New Hampshire Avenue, Silver Spring, MD 20993 USA

**Keywords:** B lymphocytes, Antibodies, Generation of diversity, Receptor editing, Rodent, Tonic signaling

## Abstract

**Background:**

The naïve antibody repertoire is initially dependent upon the number of germline V(D)J genes and the ability of recombined heavy and light chains to pair. Individual VH and VL genes are not equally represented in naïve mature B cells, suggesting that positive and negative selection also shape the antibody repertoire. Among the three member murine Vκ10 L chain family, the Vκ10C gene is under-represented in the antibody repertoire. Although it is structurally functional and accessible to both transcriptional and recombination machinery, the Vκ10C promoter is inefficient in pre-B cell lines and productive Vκ10C rearrangements are lost as development progresses from pre-B cells through mature B cells. This study examined VH/Vκ10 pairing, promoter mutations, Vκ10 transcript levels and receptor editing as possible factors that are responsible for loss of productive Vκ10C rearrangements in developing B cells.

**Results:**

We demonstrate that the loss of Vκ10C expression is not due to an inability to pair with H chains, but is likely due to a combination of other factors. Levels of mRNA are low in sorted pre-B cells and undetectable in B cells. Mutation of a single base in the three prime region of the Vκ10C promoter increases Vκ10C promoter function in pre-B cell lines. Pre-B and B cells harbor disproportionate levels of receptor-edited productive Vκ10C rearrangements.

**Conclusions:**

Our findings suggest that the weak Vκ10C promoter initially limits the amount of available Vκ10C L chain for pairing with H chains, resulting in sub-threshold levels of cell surface B cell receptors, insufficient tonic signaling and subsequent receptor editing to limit the numbers of Vκ10C-expressing B cells emigrating from the bone marrow to the periphery.

## Background

The potential diversity of the naïve antibody repertoire is dependent in part upon the number of functional VH and VL genes that are able to undergo successful V(D)J recombination and H-L chain pairing, followed by the selection events that occur during B cell development. The murine Ig kappa locus spans over 3 Mb on chromosome six and is composed of approximately 140 Vκ genes that reside in the large five-prime end of the locus, four Jκ genes, a kappa constant region gene and three enhancers [1-6]. Of the 140 murine Vκ genes that have been mapped and sequenced [7-9], 95 are considered to be potentially functional while 45 are considered to be defective due to mutations in promoters, splice sites, recombination signal sequences, the presence of stop codons or frame shifts, and the replacement of invariant amino acids. Vκ genes can be in either the same or opposite transcriptional orientation relative to the Jκ genes, with recombination resulting in either a deletion or inversion of the intervening sequences. The frequency of Vκ gene usage does not exhibit positional bias in early development and usage in adults does not correlate with the size of a given Vκ family [10-14]. Approximately 40% of Vκ genes are in the opposite transcriptional orientation relative to Jκ and rearrange by inversion, which may affect the overall usage pattern of particular Vκ genes. Utilization of individual Vκ genes in splenic B cell cDNA libraries from BALB/c mice and in Vκ cDNA transcripts from B cells of bone marrow, spleen and lymph node of C57BL/6 mice have been shown to range from 0.001% to 12.5% [15,16] with skewing of the repertoire towards a limited set of Vκ genes and families. In one study, skewing of the repertoire by preferred Vκ-Jκ rearrangements was such that seven Vκ genes represented greater than 40% of the repertoire in C57BL/6 mice [16]. It is not known why other potentially functional Vκ genes would be underutilized, but it was hypothesized that the observed preferential Vκ gene usage could be the result of intrinsic rearrangement preferences and/or differences in expression levels.

Factors that have the potential to affect the inclusion of a V region gene in the available repertoire include changes in nuclear organization [17-19] and subsequent changes in accessibility of the Ig loci to the transcription and recombination machinery [20-29], recombination efficiency due to differences in the sequences of the recombination signal sequence (RSS) [30-32], promoter efficiency [33-35] receptor editing [36] and the ability of H and L-chains to pair [37,38].

We have utilized the murine Vκ10 family of immunoglobulin light chain genes to examine multiple factors that can influence a Vκ gene’s contribution to the antibody repertoire. The Vκ10 family contains three structurally functional members, Vκ10A (IMGT IGKV10-96), Vκ10B (IMGT IGKV10-94) and Vκ10C (IMGT IGKV10-95) that share greater than 94% nucleic acid and 91% amino acid homology. Vκ10A and B are expressed in antibody responses to a wide variety of both T-dependent and T-independent antigens, while Vκ10C has never been detected as part of a functional antibody. Furthermore, Vκ10C mRNA is present at low levels in bone marrow, spleen and lymph nodes [16,39,40]. Our previous studies of the Vκ10 family have shown that all Vκ10 genes are accessible as measured by levels of sterile transcripts and not impaired in their ability to rearrange in pre-B cells. The promoter for Vκ10C was less efficient than the Vκ10A promoter in pre-B cell lines but worked equally as well as the Vκ10A promoter in immature, mature and plasma cell lines. The frequency of cells carrying productive Vκ10C rearrangements are lost as pre-B cells develop into immature IgM^+^ cells in the bone marrow and through the transition to mature B cells in the spleen. Impaired VH and VL pairing and poor transcription of Vκ10C may lead to reduced BCR expression and insufficient tonic signaling, which may cause the loss of Vκ10C productive rearrangements by receptor editing. Additionally, negative selection of B cells bearing Vκ10C light chains would likewise trigger receptor editing and result in loss of productive Vκ10C alleles.

In the current study, we compared levels of Vκ10 transcripts in pre-B and splenic B cells to known promoter activities in transient transfection assays and examined the Vκ10C promoter using site specific mutagenesis to identify sites responsible for promoter inefficiency. The ability of Vκ10C L chains to pair with H chains was examined using phage display and receptor editing was assessed by quantifying displacement of Vκ10 productive rearrangements by secondary VκJκ rearrangements or recombining sequence (RS) editing events. Our results suggest that a combination factors result in the underutilization of the Vκ10C gene in the naïve antibody repertoire.

## Methods

### Ethics statement

BALB/cAnCr mice were purchased from the National Cancer Institute, Division of Cancer Treatment, Frederick, MD. Mice were maintained and experiments were performed in accordance with the Center for Biologics Evaluation and Research Animal Care and Use Committee regulations under protocol WO-2012-60.

### Pre-B cell sorting and cDNA synthesis

Bone marrow was collected from femurs of 12–16 week old male and female BALB/cAnNCr mice (National Cancer Institute, Division of Cancer Treatment, Frederick, MD). Red cells were lysed with ACK lysis buffer for 3 min on ice, and the remaining cells were washed in PBS and resuspended at 2 ×10^7^ cells/ml in FACS buffer. Cells were stained with PE-anti-B220 (BD Pharmingen, San Diego, CA), FITC anti-mouse IgM (BD Pharmingen) and biotin anti-CD43 (BD Pharmingen) for 30 min on ice. After two washes, cells were stained with streptavidin-PE (Molecular Probes, Eugene, OR) for 30 min on ice. Cells were resuspended at a concentration of 1.5 × 10^7^ cells/ml and sorted on a Becton Dickinson FAC-Star^Plus^ for IgM^−^/B220^+^/CD43^−^ pre-B cells. Seven individual sorts were performed, each with cells from 3–4 mice. Two micrograms (μg) of RNA purified from the pre-B cells using the Trizol method (Life Technologies, Carlsbad, CA) was used to synthesize cDNA using the Omniscript (Qiagen, Venlo, Netherlands) protocol with either oligo dT or the gene-specific CK73 primer (5′-cctgttgaagctcttgacaatgggtg-3′), which binds near the 3′ end of the kappa constant region. One tenth (2 μl) of each cDNA reaction was used for each Vκ10A, B or C quantitative PCR (qPCR) reactions as described below.

### Vκ10 qPCR in pre-B cells and spleen

Vκ10A, B and C qPCR standard templates were made by PCR using total BALB/c spleen cDNA. RNA was made from 5 × 10^6^ BALB/c spleen cells using the Trizol method and cDNA was synthesized using the Omniscript protocol and an oligo dT primer. Primers for amplification of Vκ10 standards include the gen11 primer (5′-tcctccctgtctgcctctctggg-3′), which resides in framework 1 and is identical in all three Vκ10 genes, and the CK73 primer which anneals to the kappa constant region. One tenth (2 μl) of the spleen cDNA was PCR-amplified in a 100 μl reaction containing 1.5 mM MgCl_2_, 0.05 mM dNTP’s, and 50 pmol of each primer. PCR reaction conditions consisted of 95°C for 4 min, 95°C for 1 min/72°C for 2 min (30 cycles), 72°C for 10 min, and a 4°C hold. PCR products were ligated into the pCR 4.0 TOPO vector (Life Technologies, Carlsbad, CA) and used to transform TOP10 *E. coli*. Transformants were screened for Vκ10 A, B or C plasmids by colony lift and blotting with ^32^P-labeled Vκ10A, B or C-specific oligonucleotides as previously described [39]. Isolated plasmids containing Vκ10A, B and C rearrangements to Jκ1 were verified by sequencing and used as standards for qPCR.

Quantitative PCR’s for each Vκ10 gene utilized the Gen11 upstream primer, the downstream primer Vκ10FW3Rev1 (5′-agacccactgccactgaaccttgatg-3′) and unique FAM-TAMRA probes for Vκ10A (5′-taaataattgctaatgtcctgacttgccct-3′), Vκ10B (5′-taaataattgctaatgccctgacttgcact-3′) and Vκ10C (5′-taaataagtgctaatgtcctcacttgccct-3′). The Gen11 and Vκ10FW3Rev1 primers bind to identical sites in all three Vκ10 genes while the unique probes differ from each other by several bases.

Vκ10A, B and C targets from sorted pre-B cell and whole spleen cDNA were quantified relative to dilutions of the Vκ10A, B and C plasmid standards. Each Vκ10 standard was diluted from 2 × 10^6^ femtograms (fg) per reaction to 2 fg per reaction. Reaction plates included triplicate standard dilutions, duplicate cDNA samples and cross-detection controls to ensure that each probe was specific for its target. Threshold cycles (Ct) for each dilution of standard were used to construct a standard curve and sample concentrations were determined by interpolation of sample Ct into the standard curves.

### Vκ10 promoter reporter vector construction, transfection and luciferase assays

Construction of the pGL3 vectors containing only the κ enhancer (κen) and the Vκ10A (AS) or Vκ10C (CS) short/minimal promoter fragments plus the kappa enhancer were previously described [40]. The Vκ10C mutated promoters S1, S2 and S3 were synthesized by site directed mutagenesis of the pGL3κenCS plasmid using the Transformer Site-directed Mutagenesis kit (Clontech, Mountain View, CA). Mutated promoters from the site mutation reactions were subsequently PCR-amplified and ligated into pGL3κen. Orientation and promoter sequence confirmation were determined by sequencing. The Vκ10C promoter was mutated such that a specific Vκ10C nucleotide was changed to that of Vκ10A. The S1 mutation introduces an additional “A” upstream of the octamer in Vκ10C, S2 introduces a C → G mutation in the 5′ E-box and S3 introduces a C → A mutation in the 3′ region of the promoter. All plasmids, including the control vector pCMV-β, (Clontech, Palo Alto, CA) were purified by double-banding in cesium chloride.

Transfections, lysate production and luciferase and β-galactosidase assays were performed as previously described [40]. Briefly, NFS-5 and 18–81 pre-B cells were co-transfected with pCMV-β and either the AS, CS, S1, S2, S3 or κen vectors and cultured for 24 hours. After 24 hours, cells were harvested, lysed and assayed for luciferase and β-galactosidase production on a luminometer in 96-well Microlite I plates (Dynatech, Chantilly, VA). Luciferase activity was normalized by dividing the luciferase value by the β-galactosidase value for each well. Statistical comparisons were performed using a two sample T-test with unequal variance.

### Construction and screening of Vκ10/HC phage libraries for pairing analysis

Full length Vκ10A, Vκ10B and Vκ10C genes rearranged to Jκ1 were obtained by RT-PCR of BALB/c spleen cDNA. The five-prime Vκ10 primers spanned codons 1–10 and included *Xho*I restriction sites (Vκ10A/B *Xho* 5′-ctccaggtcgacctcgaggatatccagatgacacagactacatcctcc-3′, Vκ10C *Xho* 5′-ctccaggtcgacctcgaggatatccagatgacacagactacttcctcc-3′). The three-prime Cκ*Asc*I (5′-tagaataggcgcgccttattatctaacactcattcctgttgaagc-3′) primer was specific for the last six codons of the kappa constant region and contained an *Asc*1 restriction site. Amplified Vκ10A/B/C/Jκ1Cκ PCR products were first cloned into the pCR4-TOPO vector. pCR4-TOPO Vκ10 clones were subsequently digested with *Xho*I and *Asc*I and purified inserts were ligated into similarly digested pCES phagemid vector [41]. Vκ10 sequences were confirmed by sequencing.

Heavy chain VDJ-CH1 rearrangements were amplified from cDNA from total RNA of LPS-stimulated splenic B cells (two individual mice), sorted pre-B cells (pooled from 5 mice) and from pre-B cells expanded ex-vivo on OP-9 stromal cells stimulated with IL-7 [22]. H-chain gene repertoires were amplified from the cDNA with the FastStart Fidelity PCR System (Roche, Basel, Switzerland) in combination with framework 1 VH primers [42] containing *Sfi*I restriction sites and a reverse IgM CH1 primer (5′-gagtcattctcgactgcggccgctggaatgggcacatgcagatctctgtttttgcc-3′) containing a *Not*I restriction site. VH PCR reaction conditions were 95°C for 5 min, 95°C for 1 min/50°C 1 min/72°C 1 min (35 cycles), 72°C for 7 min, and a 4°C hold. H-chain PCR products were digested with restriction enzymes *SfiI* and *Not*I and gel-purified. 350 ng of the purified H-chain DNA PCR products were ligated into 1 μg of gel-purified, *SfiI-* and *Not*I-digested pCES phagemids containing a fixed Vκ10 A, B or C Jκ1-Cκ gene with 10 μl T4 Ligase (Life Technologies, Carlsbad, CA) in a 200 μl reaction overnight at room temperature. Ligations were precipitated, resuspended in 15 μl of H_2_O and electroporated into 300 μl of *E. coli* strain XL-1 Blue (Stratagene, La Jolla, CA) in 2 mm cuvettes (BioRad, Hercules, CA) with a BioRad Gene pulser set at 2.5 kV, 200 Ω, and 25 μF. Electroporated cells were added to 5 ml of LB/ampicillin, grown for one hour at 37°C and the entire library was spread onto LB/ampicillin plates and incubated overnight at 37°C. Colonies were scraped from the plates and saved as glycerol stocks. In total, 10 libraries were generated, each with a complexity of ~1×10^7^ total cfu.

Forty μl of each library was used for library expansion and subsequent phage rescue using M13K07 helper phage. Phage expressing Vκ10/H chain F_Ab_ were rescued from each library, PEG-precipitated and resuspended in 1 ml PBS. Each phage library was subjected to two rounds of selection, first on 96-well plate wells (two wells) coated with 500 ng goat anti-κ antibodies (Southern Biotechnology, Birmingham, AL) and second on two wells coated with 500 ng goat anti-μ antibodies (Southern Biotechnology, Birmingham, AL). For each selection, wells of a 96 well plate were coated with antibody overnight at 4°C in coating buffer (sodium bicarbonate buffer) and then blocked with 300 μl/well of 1% BSA/PBS for one hour at 37°C. Fifty μl of freshly prepared phage in PBS was added to each well (2 wells per library) and incubated for two hours at 37°C. Wells were washed ten times with 300 μl PBS/0.05% Tween 20, bound phage were eluted with 50ul of 100 mM glycine-HCl pH 2.2 and then neutralized with 3 μl of 2 M Tris. Ten μl of neutralized phage was then used to re-infect XL-1 Blue cells. Phage were rescued and purified as described above and the selection process was repeated on anti-μ coated wells. XL-1 Blue cells were infected with phage eluted during the second round of selection and plated on LB/ampicillin plates. Phagemid DNA from approximately 300 individual bacterial colonies per library were purified and sequenced. Sequences were compared against the existing VH sequence data for the C57BL/6 and 129S1 strains.

### PCR of Vκ10Jκ1 displaced by secondary rearrangements to Jκ2

Genomic DNA was isolated from BALB/c spleen cells and sorted pre-B cells and PCR was performed using primers designed to amplify Vκ10 rearrangements that were displaced, but retained in the genome by secondary inversional Vκ gene rearrangements to Jκ2. Vκ10Jκ1 products were amplified with the Gen 9 primer (5′-tccagatgacacagactac-3’), which binds the identical sequence in framework 1 of all Vκ10 genes and the Jκ2HepNanR 3′ primer (5′-gbytgwakcactgtgcacagtggtgtcccttc actca-3′) that includes a portion of the Jκ2 RSS 23 bp spacer, the back-to-back Vκx/Jκ2 heptamers and part of the RSS 12 bp spacer consensus of Vκ genes. The sequence of the 12 bp Vκx spacer portion of the primer was designed by construction of a consensus sequence from published Vκ gene sequences [7-9]. PCR conditions were as follows: 95°C for 5 min, 95°C for 30 sec, 55°C for 30 sec, 72°C for 1 min, repeated for 35 cycles, with a final extension of 72°C for 7 min. PCR products were ligated into the pCR4-TOPO vector and 2 μl of the ligation reactions were transformed into TOP10 *E. coli* cells. Two-hundred randomly chosen clones from each transformation were grown overnight, purified with Montage Plasmid Miniprep Kit (Millipore, Billerica, MA) kit and sequenced.

### PCR of VκJκ-iRS recombination

Genomic DNAs were isolated from spleens of 4 BALB/cAnNCr mice using the Genomic Prep Cells and Tissue DNA Isolation Kit (Amersham Pharmacia Biotech, Piscataway, NJ) or Trizol according to manufacturers’ instructions. PCR included the Gen 9 five-prime primer and the three-prime RS-101 primer (5′-acatggaagttttcccgggagaatatg-3′), which anneals in the RS sequence [43]. Six replicate PCRs were performed using the GeneAmp XL PCR kit (Life Technologies). Each reaction contained 100 ng genomic DNA; 800 μM Mg(OAc)_2_; 2 units rTth DNA polymerase XL; 200 μM each dNTP and 20 picomol each primer. Amplification was performed in a DNA Thermal Cycler 480 (PE Biosystems, Foster City, CA) at 93°C for 1 minute, followed by 35 cycles of 93°C for 1 minute, 59°C for 1 minute, and 72°C for 2.5 minutes with final extension for 10 minutes at 72°C. Replicate PCR products were pooled, precipitated, and ligated into pCR2.1-TOPO vector (Life Technologies, Carlsbad, CA).

## Results

### The Vκ10C promoter is inefficient in pre-B cells

**Figure 1:**
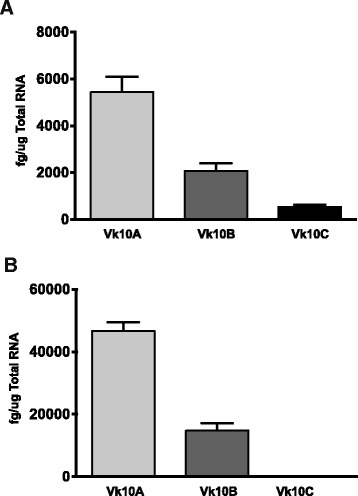
**Vκ10 quantitative RT-PCR. (A)** BALB/c IgM^−^/CD43^−^/B220^+^ bone marrow cells and **(B)** spleen. A maximum of two μg RNA was used as template for RT reactions with oligo d(T) or Cκ primers. One tenth of each cDNA reaction was used as template in qPCR with primers that amplify all Vκ10 genes equally. Gene-specific probes were used in each reaction to identify Vκ10A, B or C. Vκ10A, B and C standard templates were used as cross-probe controls in each experiment and dilutions were used to construct standard curves. The level of Vκ10 target in each sample was calculated by interpolation into the standard curve. Results are expressed as femtograms (fg) of detected Vκ10 target/μg of total RNA in the RT reaction.

We assessed levels of Vκ10 transcripts in freshly isolated pre-B cells and spleen using qPCR. Vκ10C mRNA levels in IgM^−^/CD43^−^/B220^+^ pre-B cells from 4 independent sorts are 4–10 fold lower than Vκ10A and Vκ10B, respectively (Figure 1A). Vκ10A and Vκ10B levels in the spleen were elevated 7–8 fold compared to levels in pre-B cells, but Vκ10C was not detected (Figure 1B).

**Figure 2:**
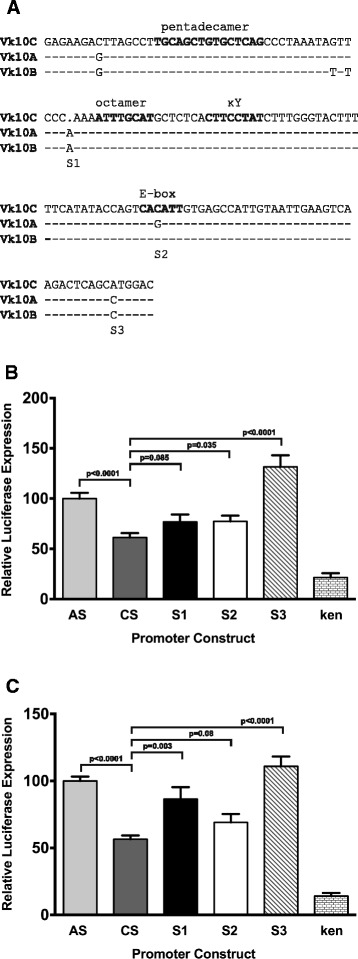
**Vκ10 promoter efficiency in pre-B cells. (A)** Nucleic acid sequence comparison of the Vκ10 promoters. S1, S2 and S3 indicate the positions in the Vκ10C promoter that were mutated to the Vκ10A sequence. Mutated Vκ10 promoters were cloned into the PGL3 luciferase reporter vector containing the κ intronic enhancer (κen). Known regulatory sequences are in bold. PGL3κenAS, PGL3κenCS, PGL3κenS1, PGL3κenS2, PGL3κenS3 and PGL3κen were co-transfected with the control vector pCMV-β into **(B)** NFS-5 pre-B cells in 3 separate experiments (n = 15 for AS, CS, S1, S2, S3, n = 9 for κen) and **(C)** 18–81 pre-B cells in 7 separate experiments (n = 33 for AS, CS, S1, S2, S3, n = 17 for κen). Luciferase expression was assayed after 24 hours and differences in Vκ10A and Vκ10C promoter-driven expression of luciferase were compared by T-test. Error bars represent standard error of the mean. AS and CS are abbreviations for the Vκ10A and Vκ10C short/minimal promoter sequence.

We next examined the effect of mutations in the Vκ10C promoter to drive luciferase expression in transient transfection assays. The Vκ10A and Vκ10C promoters differ by four nucleotides over an approximately 130 bp region (Figure 2A). In luciferase reporter gene assays, the Vκ10C promoter has a significantly lower efficiency than the Vκ10A promoter in the pre-B cell lines 70Z/3, NFS-5, NFS-467, and 18–81, but is equally efficient in immature, mature and plasma cell lines [40]. Here, we introduced single mutations at three positions into the Vκ10C promoter to resemble the Vκ10A promoter and placed each mutated promoter upstream of the luciferase reporter gene in pGL3κen [40], which contains the kappa intronic enhancer downstream of the luciferase reporter gene. We did not make the C → G mutation upstream of the pentadecamer as the germline sequence available for Vκ10A at the time did not show this difference.

Promoter efficiency of the mutated Vκ10C promoters at site 1, site 2 and site 3 (S1, S2, S3) was assessed in transient transfection assays using NFS-5 and 18–81 pre-B cell lines. The unmutated short/minimal Vκ10A (AS) and Vκ10C (CS) promoters and a vector containing only the kappa enhancer (κen) were included as controls. As expected, a highly significant difference (p < 0.0001) was observed between the activity of the unmutated AS and CS minimal promoters in NFS-5 (Figure 2B) and 18–81 (Figure 2C) cells, while vector containing only the kappa enhancer exhibited little activity. By mutating the site 3 (S3) nucleotide (A → C, at the 3′ end of the promoter), luciferase expression was consistently and reproducibly increased in pre-B cells to a level comparable to that attained with the AS vector (p < 0.0001, Figure 2B and C). Mutations to site 1 (S1; upstream of the octamer) and site 2 (S2; 5′ E-box) marginally improved promoter function , but did not yield consistently reproducible results between the two pre-B cell lines (Figure 2B and C). Together, these data suggest that the Vκ10C promoter is less efficient than the Vκ10A promoter in pre-B cells and pre-B cell lines, specific sequences control promoter efficiency, and splenic B cells expressing Vκ10C L chains are rare so that Vκ10C transcripts cannot be detected.

### Vκ10C L chain pairs efficiently with different H chains

The ability of Vκ10 L chains to pair with a diverse array of H chains was tested in a phage display format that expresses F_Abs_. The H chain is expressed as a fusion protein with the phage coat protein gIII, while the L chain is soluble and pairs with the H chain in the periplasmic space to form the F_Ab_; hence, only Vκ10 L chains that can successfully pair with H chains would be expressed on the phage surface. Two libraries for each Vκ10 gene were generated from LPS-stimulated splenic B cells from two mice. The data from both libraries for each Vκ10 gene were pooled for analysis. A pool of sorted pre-B cells from 5 mice was used to generate a pre-B cell library for each Vκ10 gene. One additional Vκ10C library was generated from a single mouse (also used to construct the second set of B cell libraries) whose pre-B cells were expanded *ex-vivo* on OP9 stromal cells stimulated with IL-7 [22]. The results from the two Vκ10C pre-B cell libraries were pooled for analysis. The H chain sequences obtained from each library were identified by comparison with germline H chain gene sequences in the IgBLAST database [44]. BALB/c mice share a VH haplotype with 129S1 mice and H chain sequences from the 129S1 strain have been reported for all but the most 5′ H chain families [45]. Accordingly, the majority of our H chain sequences show the greatest homology to 129S1 H chain sequences while others are identified using germ line H chain gene sequences from different strains in the database.

**Figure 3:**
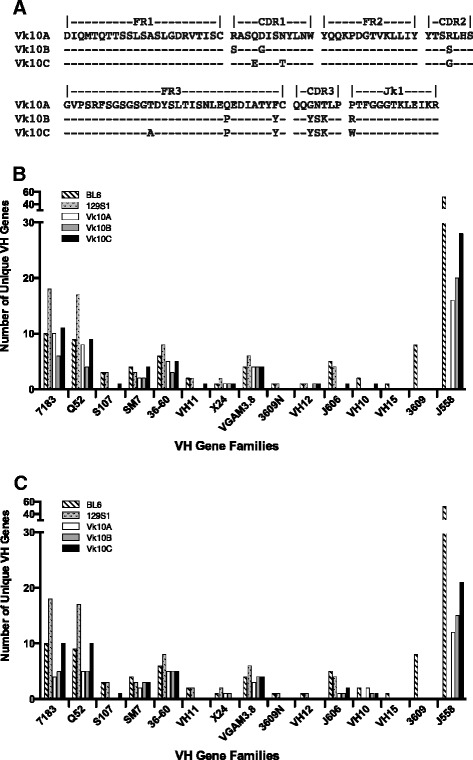
**Vκ10 light chain pairing with heavy chain. (A)** Alignment of the Vκ10AJκ1, Vκ10BJκ1 and Vκ10CJκ1 amino acid sequences derived from rearrangements amplified by PCR and cloned separately into the phagemid vector pCES (Cκ is not shown but is identical for all). **(B)** VH genes amplified from pre-B cells and **(C)** VH genes amplified from splenic B cells. VDJ-CH1 rearrangements were amplified from cDNA derived from total RNA of LPS-stimulated splenic B cells (two mice separately), sorted pre-B cells (pooled from 5 mice) and from pre-B cells expanded ex-vivo on OP-9 stromal cells stimulated with IL-7. Phage expressing both μ and κ were selected by sequential panning on anti-κ and anti-μ coated surfaces and sequenced. The genomic linear order of V_H_ gene families from five prime to three prime is shown on the bottom of each graph. Bars with diagonal lines and gray stippled bars represent the number of germ line V_H_ sequences in each V_H_ family for C57BL/6 and 129S1 mice, respectively. White, gray and black bars show the number of unique V_H_ sequences isolated for each V_H_ family that paired with Vκ10A, Vκ10B and Vκ10C, respectively.

Roughly three hundred clones from each library (3000 total) were sequenced to allow an observation of an event occurring at a frequency as low as 1%. Figure 3A depicts the fixed Vκ10Jκ1 sequences used in the pairing experiments. The number of individual V_H_ chains per V_H_ family that were paired with Vκ10A, Vκ10B and Vκ10C in pre-B cell and splenic B cell libraries are shown in Figure 3B and C, respectively. In the pre-B cell libraries (Figure 3B), Vκ10C paired with V_H_ chains from 12 of 15 V_H_ gene families (67 unique VDJ sequences), while Vκ10A paired with V_H_ chains from 7 families (46 unique VDJ sequences) and Vκ10B paired with V_H_ chains from 8 families (41 unique V sequences). A diverse pattern of pairing for all three Vκ10 family members was also observed in the splenic B cell libraries (Figure 3C). Here Vκ10A, Vκ10B and Vκ10C L chains each paired with V_H_ chains from 9 different V_H_ families using 35, 40 and 57 unique VDJ sequences, respectively. These data demonstrate that Vκ10C is not deficient in its ability to pair with V_H_ chains and in fact, exhibits a broader spectrum of pairing with unique V_H_ chains than the other members of the Vκ10 family in both pre-B and B cells.

### Productive Vκ10C rearrangements are eliminated by receptor editing and RS recombination

**Figure 4:**
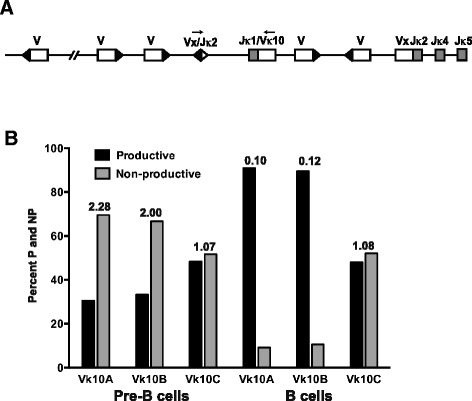
**Editing of Vκ10Jκ1 rearrangements by secondary Vκ inversion type rearrangements to Jκ2. (A)** Schematic of Vκ10Jκ1 rearrangement displaced by subsequent inversional secondary Vκ rearrangement to Jκ2. Open boxes represent Vκ genes, black triangles next to open boxes represent Vκ RSS sequences, grey boxes represent Jκ genes and the back-to-back open and black triangle represent the back-to-back Jκ2 and Vκx RSS, retained on the genomic DNA. Arrows represent the PCR primers used to amplify the displaced Vκ10Jκ1 rearrangement. The upstream primer anneals in Vκ10 framework 1 to a sequence that is identical for all Vκ10 genes. The downstream primer was designed to include a portion of the Jκ2 23 bp spacer, the back-to-back Vκx/Jκ2 heptamers and part of the 12 bp space consensus sequence of other Vκ genes. **(B)** Percent of productive and nonproductive recombination products of displaced Vκ10A, Vκ10B and Vκ10C in BALB/c pre-B cells and B cells. The NP/P ratio is listed above each set of bars.

We next examined the possibility that the loss of productive Vκ10C rearrangements is the result of editing by secondary recombination. Secondary recombination can replace productive and non-productive VκJκ recombinations via deletion or inversion, depending on the transcriptional orientation of the Vκ gene relative to Jκ [46]. Inversion events result in the retention of the original VκJκ rearrangement in the genomic DNA upstream of the back-to-back RSS structure derived from the secondary recombination (Figure 4A). The presence of these edited primary rearrangements does not influence the selection events leading to the ultimate fate of the B cell, which is based on the expressed antibody, but reflects the recombination history of the B cell. Rearrangement in only one of three reading frames would be productive and the other two reading frames would be nonproductive (a non-productive to productive ratio of 2:1). If the loss of productive Vκ10C rearrangements is largely due to negative selection resulting in receptor editing, we would expect a greater percent of in-frame Vκ10CJκ displaced by secondary rearrangements. PCR was performed to amplify primary Vκ10Jκ1 rearrangements that were displaced by secondary inversional Vκ gene rearrangements to Jκ2. In-frame and out of frame rearrangements of all three Vκ10 genes were detected in B and pre-B cell genomic DNA (Figure 4B).

In both pre-B cells and splenic B cells the majority of total Vκ10 rearrangements were Vκ10A, followed by Vκ10C and Vκ10B (Table 1). In pre-B cells, the non-productive (NP) to productive (P) recombination ratio for displaced Vκ10A and Vκ10B rearrangements was the expected ratio of ~2.0, while the NP/P ratio for Vκ10C was ~1.0, indicating equal displacement of non-productive and productive Vκ10CJκ1 rearrangements. In B cells, the NP/P ratios of displaced Vκ10A and Vκ10B rearrangements is <1.0, but remains ~1.0 for Vκ10C. These data suggest that there may be some negative selection of primary Vκ10C rearrangements as reflected by the NP/P ratio in both pre-B and B cells. For Vκ10A and Vκ10B, the NP/P ratio in pre-B cells reflects the expected outcome of primary recombination events, but the low NP/P ratios seen in B cells suggests higher rates of negative selection occurring between the pre-B cell stage and the mature B cell population.

**Table 1:** **Vκ10 editing by secondary rearrangements to VκJκ2**

	**VκJκ2 editor**				**VκJκ-iRS**			
	**Total**	**Non-productive**	**Productive**	**NP/P** ^**a**^	**Total**	**Non-productive**	**Productive**	**NP/P** ^**a**^
**Pre-B**								
**Vκ10A**	82	57	25	2.28	ND	ND	ND	ND
**Vκ10B**	36	24	12	2.00	ND	ND	ND	ND
**Vκ10C**	58	30	28	1.07	ND	ND	ND	ND
**Spleen**								
**Vκ10A**	77	7	70	0.1	58	49	9	5.4
**Vκ10B**	19	2	17	0.12	19	18	1	18
**Vκ10C**	25	13	12	1.08	69	36	33	1.09

^a^NP/P = non-productive to productive recombination ratio.

ND = not done.

**Figure 5:**
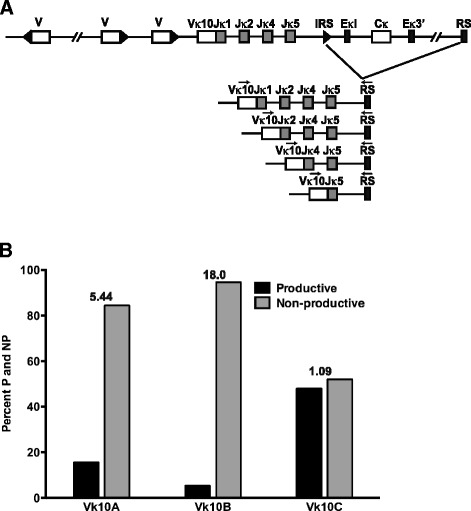
**VκJκ-iRS receptor editing of Vκ10 productive and nonproductive rearrangements. (A)** Schematic of the kappa locus (not drawn to scale) depicting VκJκ-iRS recombination products resulting from recombination of the RS element with an isolated heptamer in the Jκ-Cκ intron. Arrows represent the PCR primers used to amplify the edited Vκ10Jκ rearrangement. The upstream primer anneals in Vκ10 framework 1 to a sequence that is identical for all Vκ10 genes. The downstream primer anneals to a sequence in the RS. **(B)** Percent of productive and nonproductive recombination products of RS edited Vκ10A, Vκ10B and Vκ10C rearrangements to Jκ1, Jκ2, Jκ4 and Jκ5.

Recombination to RS is an alternate mechanism of receptor editing. VκJκ rearrangements are retained on alleles when the recombination is between an isolated heptamer in the Jκ-Cκ intron and the RS element 25 kb downstream of Cκ (VκJκ-iRS; Figure 5A). The NP/P ratios for Vκ10 rearrangements on VκJκ-iRS alleles in splenic B cells were ~1 for Vκ10C and > > 2 for Vκ10A and Vκ10B (Table 1, Figure 5B). The Vκ10C data are consistent with the secondary recombination data, suggesting some negative selection of productive Vκ10C recombination products.

## Discussion

The primary naïve antibody repertoire is shaped by events that occur during B cell development in the bone marrow as well as events that happen once an immature B cell emigrates from the bone marrow and transitions to a mature follicular, marginal zone or B1 B cell. Transgenic and knock-in mouse models expressing antibody genes have been invaluable in demonstrating a primary role for receptor editing in the establishment of central B cell tolerance. When opportunities for receptor editing are limited, newly formed B cells in the bone marrow can be rendered anergic or undergo deletion. It’s been estimated that fewer than half of newly formed B cells emigrate from the bone marrow each day [47]. The fate decisions of developing B cells are influenced by levels of BCR expression and tonic signaling, where a reduction in basal levels of tonic signaling leads to impaired maturation of pre-B cells to immature B cells, de-differentiation of the newly formed B cell, and light chain receptor editing, while elevated levels of tonic signaling lead to increased RAG expression and receptor editing [48-56]. A threshold of BCR expression and tonic signaling ultimately determines whether a B cell moves to the periphery or undergoes additional rounds of L chain recombination [57-59].

Our Vκ10 system offers the unique advantage of examining the factors that determine the inclusion or exclusion of a highly homologous family of Vκ LC’s in the antibody repertoire during normal development in the context of unmanipulated genetic backgrounds. The Vκ10 L chain family consists of three highly homologous structurally functional genes with dramatic differences in usage patterns [39,40] Vκ10A is highly utilized and represents between 2.9% to 12% of all Vκ gene usage in bone marrow, spleen and lymph nodes while Vκ10B is utilized to a lesser extent, representing between 1.7% and 3.5% of Vκ gene usage in these compartments [16,39]. TheVκ10C gene is rarely utilized and has been shown to be present at a frequency of 0.15% in BALB/c spleen B cell cDNA libraries [39], near or just below 1% in BL/6 bone marrow, spleen and lymph nodes [16], and over 1000-fold less than Vκ10A and B in BALB/c spleen mRNA [40]. Additionally, in naïve adult BALB/c mice, Vκ10C expression was detected once each from a total of 564 single-sorted follicular and 613 marginal zone B cells (0.17% frequency) while Vκ10A and Vκ10B were detected at frequencies of approximately 6% and 1.6%, respectively (Patel et al., unpublished data).

During the pre-B to immature B cell transition, IgM is expressed on the surface of the developing B cell and productive Vκ10C rearrangements are lost [39], which may be a consequence of imbalances in tonic signaling resulting from inefficient L chain expression or an impaired ability of Vκ10C to pair well with VH chains [48,52,57]. Indeed, at the VH locus, removal of the Eu enhancer from one allele is known to result in reduced transcription levels of a rearranged VH gene on the other allele, such that the surface density of BCR’s containing the rearranged VH gene is not sufficient to reach the tonic signaling threshold that is critical for transition of the cells into immature B cells [50]. Vκ10C-expressing B cells could also be lost through negative selection, leading to receptor editing to replace the productive Vκ10C rearrangement. To assess the possible reasons for lack of Vκ10C usage, we further examined promoter strength, its ability to pair with V_H_ chains, and hallmarks of negative selection (receptor editing, RS recombination). Overall, the data support the idea that insufficient BCR expression and tonic signaling resulting from low levels of Vκ10C transcription are likely the major reason for loss of productive Vκ10C rearrangements in developing B cells, although a very small number survive and migrate to the periphery.

Although our data support the concept of a weak Vκ10C promoter, in-silico analyses of the Vκ10 promoters by us and others [9] did not reveal differences in transcription factor binding motifs that might explain the weak Vκ10 promoter strength. There may be differences in binding of an as yet unknown transcription factor(s) to the Vκ10 promoter at site 3. Although germline Vκ10A and Vκ10C transcription rates indicate equal accessibility, subsequent epigenetic modifications to the promoters and the position of the rearranged genes in the contracted locus may influence their interaction with downstream enhancers and affect the rates of transcription.

Using an RNA knockdown approach in-vivo, reduction of Igκ transcription in the natural repertoire promotes increased levels of receptor editing whereas cells that maintained normal levels of Igκ transcripts and had reduced editing [57]. Conversely, increases in tonic signaling, through enhanced BCR signaling results in suppression of light chain receptor editing [58]. The lack of Vκ10C expression is consistent with the idea that inefficient expression of Vκ10C in pre-B cells may result in sub-threshold surface expression of BCRs on immature B cells. Insufficient tonic signaling would lead to receptor editing [48,52,59], resulting in the elimination of Vκ10C rearrangements due to secondary L chain recombination.

Productive and non-productive Vκ10C rearrangements associated with secondary Jκ2 recombination and VκJκ-iRS recombination, hallmarks of receptor editing, showed a higher proportion of productive Vκ10C rearrangements associated with these structures. For Vκ10C recombinations associated with either structure, the NP/P ratio was ~1, suggesting that productive Vκ10CJκ joints led to subsequent recombination events. For Vκ10A and Vκ10B in pre-B cells, the NP/P ratio on secondary recombination structures was the expected ratio of ~2, but in B cells and in VκJκ-iRS structures, the ratio was significantly <1, suggesting that between the pre-B cell stage through emigration to the periphery, cells expressing Vκ10A and Vκ10B L chains were lost via clonal deletion. These data suggest that there are highly elevated levels of tonic signaling for Vκ10A and Vκ10B H-L chain pairs, but only moderately elevated levels for Vκ10C pairs.

Vκ10C L chain was not defective in its ability to pair with unique VDJ H chains and in fact, paired with a wider array of H chains from both pre-B cells and splenic B cells than either Vκ10A or Vκ10B. There are qualitative differences between the Vκ10C- V_H_ chain pairs and Vκ10A and Vκ10B- V_H_ chain pairs, including more poly-reactivity among Vκ10C- V_H_ chain pairs (Aydanian et al., manuscript in preparation).

A small number of B cells that express productive Vκ10C rearrangements migrate to the periphery. This successful emigration may be due to specific Vκ10C-V_H_ chain pairs that have appropriate levels of tonic signaling. The majority of these Vκ10C-expressing B cells may undergo peripheral selection events leading to further loss of productive rearrangements (Aydanian et al., manuscript in preparation). B cells making a first encounter with a high-affinity auto-antigen in the periphery are deleted at the transition from T1 to T2 transitional B cell stage [60,61] and it is possible that Vκ10C-expressing B cells are eliminated here. Another possible fate of Vκ10C-expressing cells in the periphery is that they are rendered anergic as a mechanism of silencing self-reactive B cells [62]. Finally, Vκ10C-expressing B cells, albeit at very low levels, could become a part of the heterogeneous mature pre-immune repertoire. Studies are in progress to unambiguously identify the route of development for Vκ10C-expressing B cells.

## Conclusions

Overall, our findings suggest that the weak promoter of the Vκ10C gene acts at the pre-B cell stage to limit the amount of available Vκ10C L chain to generate a BCR. This leads to insufficient BCR expression and tonic signaling, thus triggering receptor editing of productive Vκ10C rearrangements. However, limited numbers of Vκ10C-expressing B cells migrate from the bone marrow to the periphery where additional mechanisms may further reduce Vκ10C-expressing B cells as they transition from immature to mature B cells.

## Abbreviations

ACK: Red cell lysis buffer
BCR: B cell receptor
BSA: Bovine serum albumin
cDNA: Complementary DNA
cfu: Colony forming units
Ct: Threshold cycle
DNA: Deoxyribonucleic acid
dNTP: Deoxynucleotide triphosphate
F_Ab_: Antigen-binding fragment of immunoglobulin
FAM: 6-carboxyfluorescein
fg: Femtogram
FITC: Fluorescein isothiocyanate
FO: Follicular B cell
mRNA: Messenger RNA
H-chain: Heavy chain of immunoglobulin
Ig: Immunoglobulin
Jκ: Kappa
kV: Kilovolt
LB: Luria-Bertani
L-chain: Light chain of immunoglobulin
LPS: Lipopolysaccharide
Mb: Megabase
MZ: Marginal zone B cell
ng: Nanogram
NP: Nonproductive Ig rearrangement
NP/P: Nonproductive to productive ratio
P: Productive Ig rearrangement
PBS: Phosphate buffered saline
PE: Phycoerythrin
PEG: Polyethylene glycol
qPCR: Quantitative PCR
RNA: Ribonucleic acid
RS: Recombining sequence
RSS: Recombination signal sequence
RT-PCR: Reverse transcription-polymerase chain reaction
TAMRA: Tetramethyl-6-carboxyrhodamine
Ω: Ohms
μF: Micro Faraday
μg: Microgram
μl: Microliter
μM: Micromolar
V(D)J: Variable, diversity, and joining
Vκ: Variable kappa
VL: Variable light chain
VH: Variable heavy chain
